# Characteristics of ultrasound device: a new technology for bone curettage and excavation

**DOI:** 10.1186/s40634-019-0203-7

**Published:** 2019-07-25

**Authors:** Tatsuo Mae, Ken Nakata, Tsukasa Kumai, Yasuyuki Ishibashi, Tomoyuki Suzuki, Takamitsu Sakamoto, Tomoki Ohori, Takehito Hirose, Hideki Yoshikawa

**Affiliations:** 1 Department of Orthopaedic Surgery, Osaka University Graduate School of Medicine, Japan. 2-2, Yamada-oka, Suita, Osaka, 565-0871 Japan; 2 Faculty of Sports Sciences, Waseda University, Japan. 2-579-15, Mikajima, Tokorozawa, Saitama, 359-1192 Japan; 3 Department of Orthopaedic Surgery, Hirsosaki University Graduate School of Medicine, Japan. 5 Zaifu-cho, Hirosaki, Aomori, 036-8562 Japan; 4 Department of Orthopaedic Surgery, Sapporo Medical University School of Medicine, Japan. S1-W6, Chuo-ku, Sapporo, Hokkaido 060-8543 Japan; 5 Orthopedic Products Department, OLYMPUS CORPORATION, Japan. 2951, Ishikawa-cho, Hachioji, Tokyo, 192-8507 Japan

**Keywords:** Arthroscopic surgery, Ultrasonic device, Curettage, Excavation, Bone tunnel, Roughness

## Abstract

**Background:**

Ultrasonic (US) devices are used in laparoscopic, dental, and spinal surgeries, while it is difficult to use for the joint under irrigation and perfusion solutions due to lack of power. A new US device is developed with greater voltage improvement and has been implemented in the arthroscopic field. The aim is to compare the characteristics of the US devices with the conventional ones in water.

**Methods:**

Twenty bone blocks from the porcine femur were settled in a holder in water. A 4.0 mm diameter abrader burr moved 15 mm along the long axis of the bone block in ten blocks for three times. A 4.3 mm wide curette blade powered by ultrasonic vibration was moved in the same manner in the other ten blocks. The gutter shape, including the gutter depth and the bottom angle of the gutter, and the curetted area ratio of the gutter were assessed.

Forty bones blocks from the porcine femurs were clamped with a holder in water, while the cortical bone surface must be located on the side. A 5 mm diameter drill excavated the bone along the previously-inserted guide wire to the 15 mm depth for twenty blocks. Next, the US excavation probe of 5x4mm rectangular shape was moved to the same depth in the other twenty blocks. Each ten block was cut in half along the bone tunnel and was assessed the surface roughness at three area, while the cross-sectional area (CSA) of the tunnel were measured and the ratio of the measured CSA was calculated based on an expected CSA in the remaining ten blocks for each device.

**Results:**

The depth of curettage and bottom angle were significantly smaller with the US device than with the abrader burr at all planes, while the curetted area ratio created by each device was mostly equal to the other. Surface roughness was similar in two evacuating devices except one area. CSA ratio with the US excavation device was significantly smaller than that with the drill.

**Conclusion:**

US curettage has an advantage to flatly curette bone surfaces, while a bone tunnel can be accurately created with the US device.

## Background

Arthroscopic surgeries are commonly performed on knee and shoulder joints, and the indication for those techniques is gradually spreading to the other small joints. During arthroscopic operations, bone/cartilage curettage and tunnel creation are common procedures. Curettes or arthroscopic abraders are used arthroscopically for the resection of osteophytes, subacromial decompression, and decortication. However, creating an even surface for hard bone is sometimes difficult when curetting with those devices. A drill is widely used to make a bone tunnel (i.e., in anterior cruciate ligament reconstruction), though error can occur when drilling to create an oblique tunnel. Thus, a guide wire/pin is inserted first during arthroscopic surgery before a drill is moved along the guide wire/pin. However, overdrilling also risks generating metal particles caused by friction between the wire and drill. Moreover, drilling may possibly dig the bone too much, because the diameter of rotation at the tip of the drill bit is larger than that at the attaching site to the handpiece due to centrifugal force.

An ultrasonically activated scalpel and an ultrasonic (US) cavitational aspirator are used widely for cutting and coagulation during laparoscopic surgery (Ai et al., 2018; Amaral, 1994a; Amaral, 1994b). For benign gallbladder diseases, laparoscopic cholecystectomy is a gold standard, while use of US scalpel is spreading in place of metal clipping. Ai et al. (Ai et al., 2018) compared the US scalpel with conventional metal clips for cystic duct closure in laparoscopic cholecystectomy in a meta-analysis, and concluded that the US scalpel was clinically superior to the conventional clips in some aspects, especially regarding shorter operating time and hospital stay. Vercellotti (Vercellotti, 2000) introduced a new US device to perform bone osteotomy for piezoelectric surgery in dental implantation. This US instrument can provide a precise and effective bone cut without damaging soft tissues compared to drills. However, the US devices are used only for small bone surgeries because of weak power, while larger power is required for the joint under irrigation and perfusion solutions due to fluid resistance (Vercellotti, 2004). A new US device (Pharmaceuticals and Medical Devices, etc. Act unapproved) was developed with greater voltage improvement and an optimized blade configuration design, and has been implemented in the field of arthroscopic surgery. This device will be available with two probes for curettage and excavation of bones of extremities upon declaration of conformity, product registration, or market clearance in each country’s jurisdiction, while not available in some areas. These probes attached to original handpieces are controlled by hand. However, to our knowledge, there are no reports on this US device. Thus, the objectives of this study are 1) to compare the characteristics of the US *curettage* device to those of the conventional abrader device and 2) to compare the characteristics of the US *excavation* device to those of a drill bit in water. US device curettes and excavates bone by vibration, while the conventional abrader device and the drill bit do by rotation. Our hypotheses are that the roughness of the curetted surface is smoother with the US device than the conventional abrader device and that the shape of the tunnel itself is more accurate with the US device than with the drill bit in excavation.

## Materials and methods

### Curettage

Ten intact fresh frozen porcine femurs were prepared. All samples were obtained from the food industry and no animals were killed or sacrificed for this study. Twenty bone blocks of 30 × 40 × 5 mm were cut from the diaphysis of the femurs. Each bone block was settled in the holder in water.

**Fig. 1:**
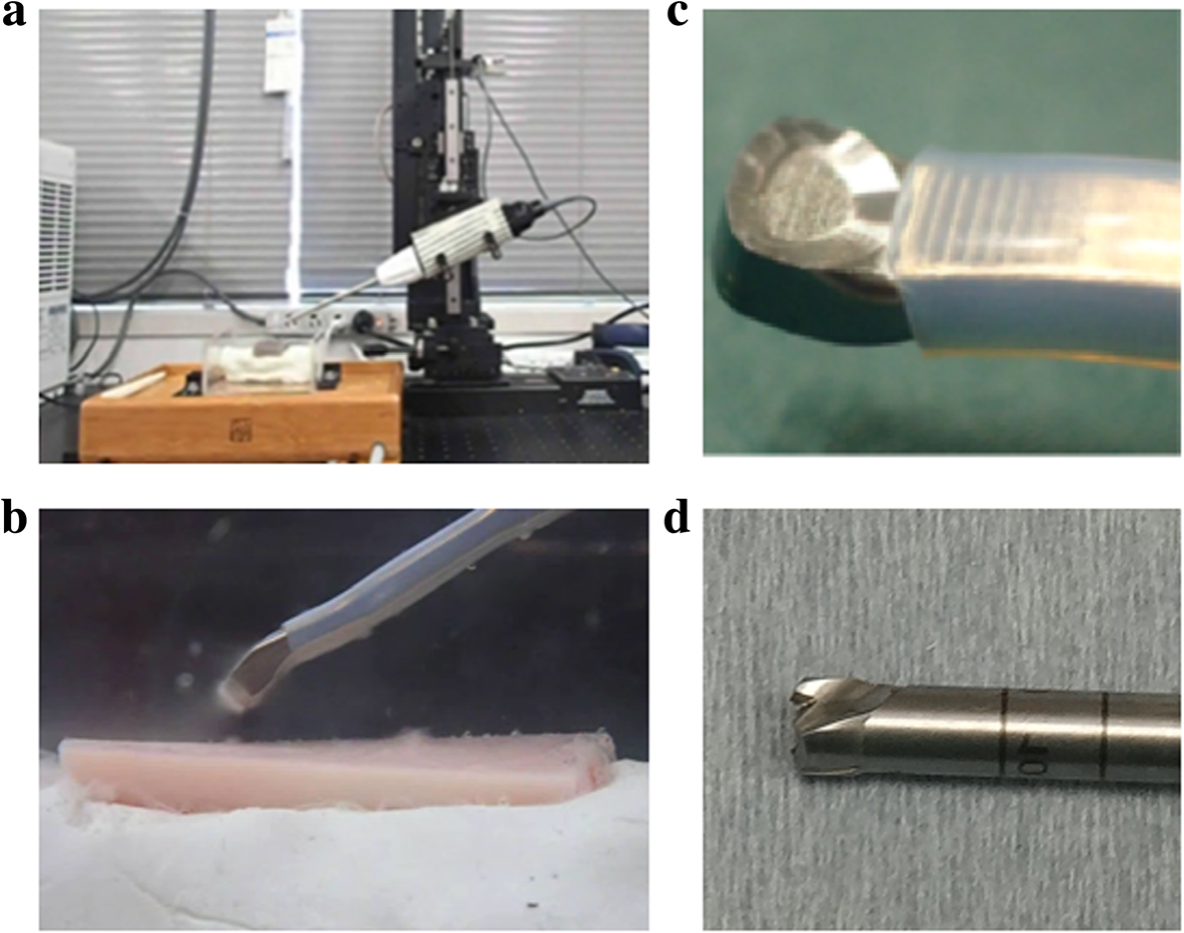
Curettage setting. **a**) Custom-made machine for bone curettage. **b**) Enlarged view of curetting. **c**) US curettage device. **d**) Tip of abrader burr

First, the φ4.0 mm Abrader (DYONICS Straight Burrs, Smith+Nephew, Andover, MA) was attached to the handpiece (DYONICS Arthroscopic Resection System, Smith+Nephew, Andover, MA), which was held with the arm in the custom-made machine. (Fig. 1) Then, the abrader burr moved 15 mm along the long axis of the bone block with a 3 N compressive load, 3.7 m/sec speed, and 30 degree of tilting angle for three times. The curetting load was preliminarily measured to ensure the clinical conditions. Two experienced orthopaedic surgeons (T.M, K.N) held the handpieces of the US and the abrader devices, which were used to curette bone on the gravimeter with clinical handling. A mean working load was then calculated as 3 N. Next, a curette blade (Olympus, Tokyo, Japan) of 4.3 mm in width was set to the US device (Olympus, Tokyo, Japan) and was moved in the same manner. The operating frequency of the US device was 47.0 kHz, the output current was 1.20 to 1.50App (Ampere peak to peak), and the highest output voltage was 1700Vpp (Voltage peak to peak).

### Evaluation of curettage

**Fig. 2:**
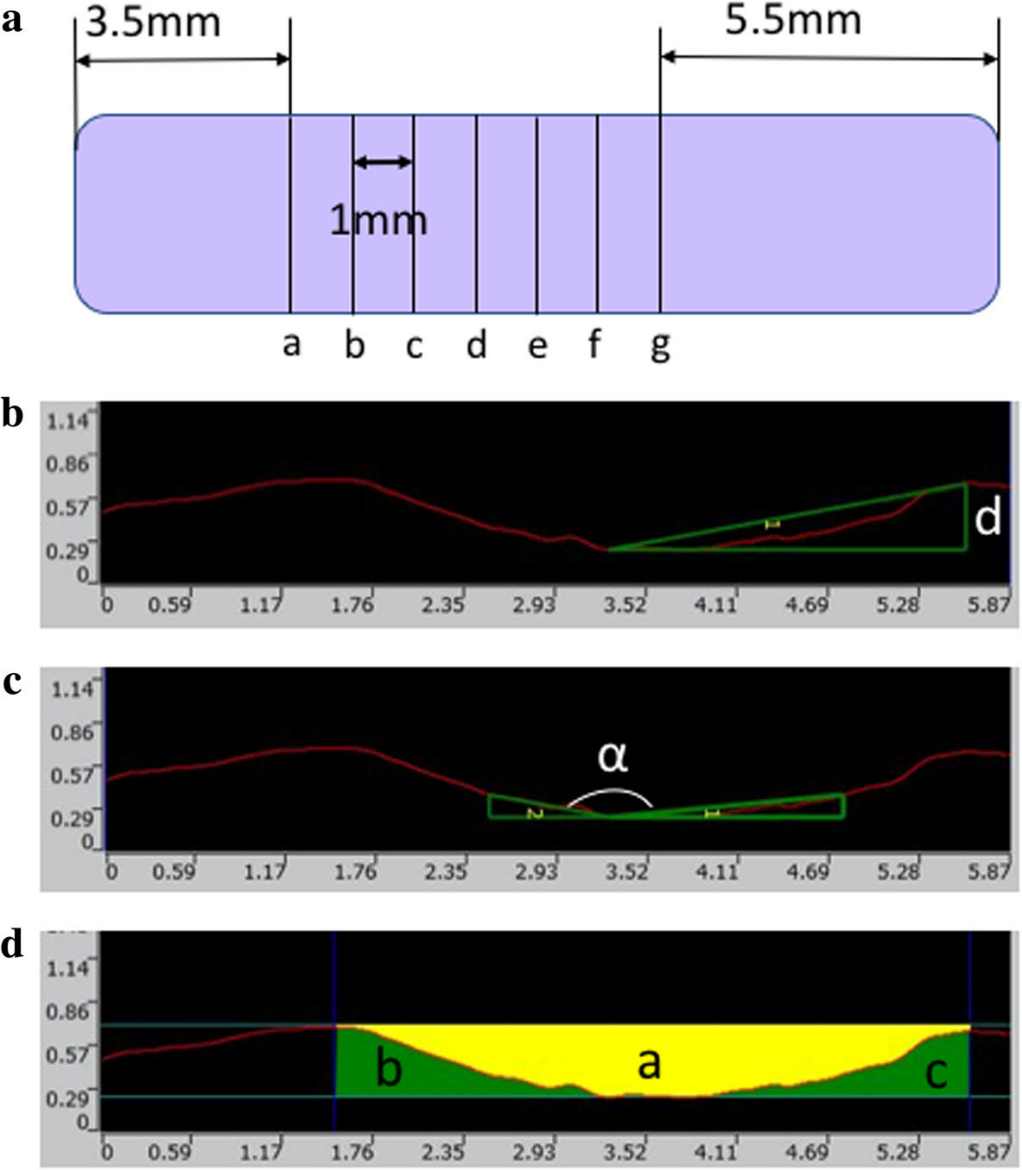
Measurement of the gutter created by the US device and the abrader burr. **a**) Measurement planes (**a-g**) to assess the shape of the gutter, started at 3.5 mm apart from the start point. *Upper* was defined as right side. **b**) Depth (**d**) of the gutter on the right side. **c**) Angle (α) of the bottom at 0.15 mm. **d**) Cross-sectional area ratio of the gutter was defined as the curetted area “a” was divided by the minimum square area “a + b + c”

The curetted surface was scanned with Opto-digital microscope (DSX500, Olympus, Tokyo, Japan) and were evaluated using DSX-BSW Application Software (ver. 3.1.1.10, Olympus). To exclude the effect of loading variation around the start and end points, the transverse planes were established every 1 mm at 3.5 to 9.5 mm away from the start point. (Fig. 2) The shape of the gutter, including depth of the gutter and angle of the bottom, and the curetted area ratio of the gutter were assessed at each plane. For the gutter depth, the distance was measured between the bottom of the gutter and the remaining surface line of one sides. The angle of the bottom was defined as the angle between the bottom lines at 0.15 mm depth. The curetted area ratio was calculated as the cross-sectional area (CSA) of the gutter was divided by that of the minimum square including the gutter. Moreover, the roughness of the curetted surface was assessed using OLS5000 Analysis application (ver. 1.2.1.116, Olympus) after photos were taken with a three-dimensional (3D) laser confocal microscope (OLS5000, Olympus, Tokyo, Japan). Roughness was defined as the difference between the average of the top line and that of the bottom line.

### Excavation

**Fig. 3:**
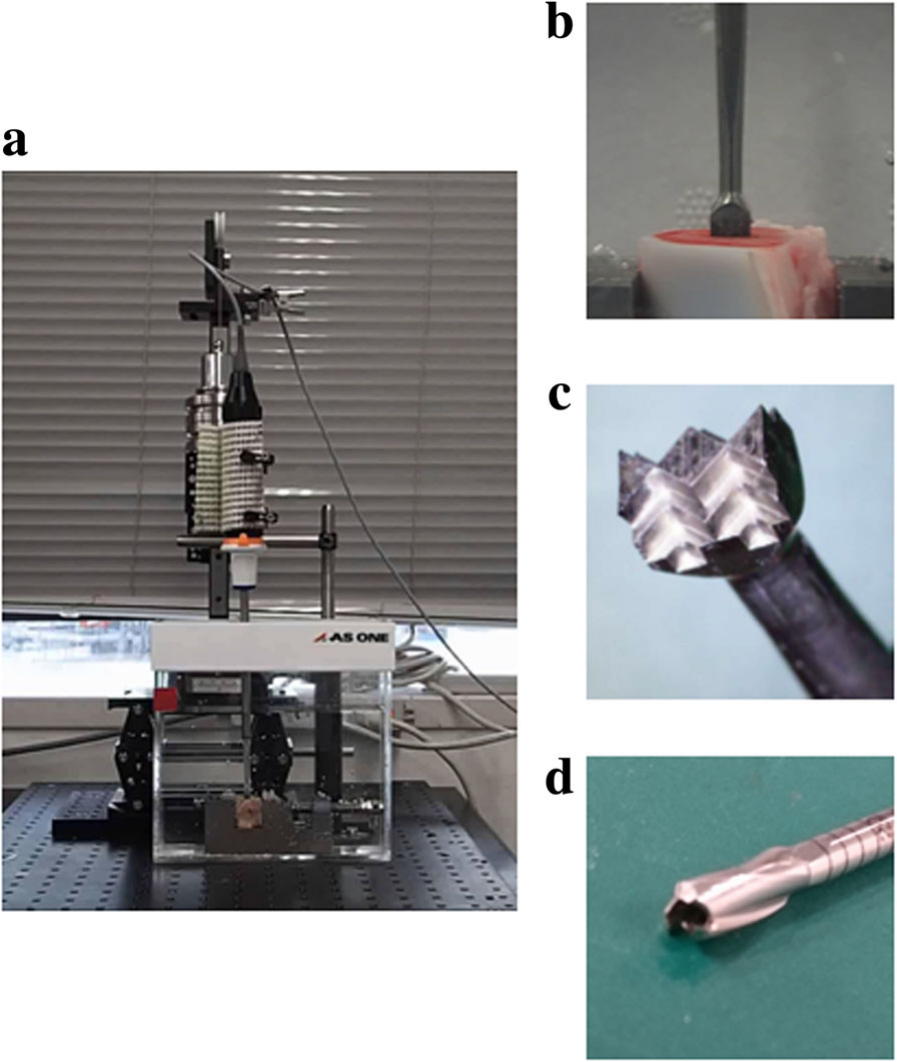
Excavation setting. **a**) Custom-made machine for bone excavation. **b**)Enlarged view of excavating. **c**) US blade of 4 × 5 mm rectangular shape. **d**) Drill bit of 5 mm in diameter

A total of 40 bones blocks (20 × 20 × 25 mm) from the proximal condyle of porcine femurs were prepared and were clamped with the holder in water. (Fig. 3) The cortical bone surface must be located on the side so that the excavation could be performed through the cancellous bone. Before excavation, microfracture was always performed at the center of the upper side of the bone block with a microfracture awl, following the clinical situation. Those blocks were then divided into two groups (drilling or US).

First, the handpiece of drill bit was attached to the arm in the other custom-made machine. A 2.4-mm Kirschner wire (K-wire; Mizuho, Tokyo, Japan) was inserted at the previously-microfracutred point of the bone to the depth of over 15 mm. Then, over-drilling was performed with a 5 mm diameter drill (Smith+Nephew, Andover, MA) along the K-wire to the depth of 15 mm under a 15 N load. This excavation load was also previously measured as two orthopaedic surgeons (T.M, K.N) clinically drilled a bone on the gravimeter. Next, the US device (Rectangular Blade; Olympus, Tokyo) was attached to the arm in the machine, and the tunnel of 15 mm in length was created along the long axis. The tip shape of the US device was 5 × 4 mm of rectangular shape. Anatomic rectangular tunnel anterior cruciate ligament (ACL) reconstruction with a bone-patellar tendon-bone graft, which was a gold standard for graft, could create bone tunnel ideally within anatomical ACL attachments and is widely performed with good outcomes (Shino et al., 2005; Shino et al., 2008; Tachibana et al., 2018). Thus, the rectangular shape probe was firstly developed.

### Evaluation of excavation

**Fig. 4:**
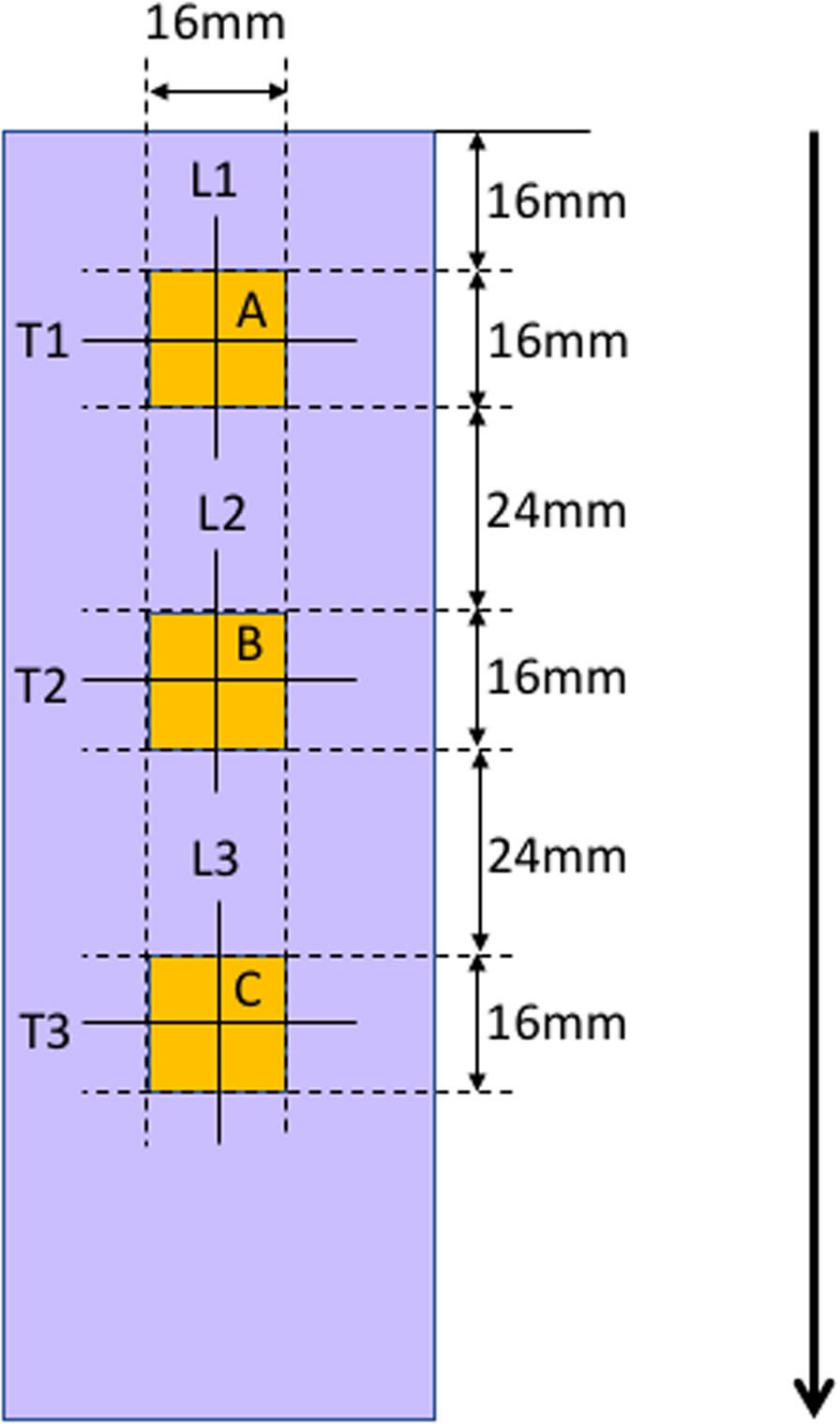
Roughness of tunnel wall was assessed at three areas. Drill and US device were moved from the upper side to the lower side

Twenty bone blocks (10 bone blocks for each device) were cut in half along the bone tunnel, remaining on the wall of the longer side. Photos of the tunnel wall were taken with the 3D laser confocal microscope (OLS5000, Olympus, Tokyo) and were assessed using the OLS5000 Analysis application (ver. 1.2.1.116, Olympus). Surface roughness of a 1.6 × 1.6 mm square at three areas at 2.4 mm intervals was calculated, whereas longitudinal and transverse linear roughness were also assessed on the center line of each side of the 1.6 × 1.6 mm square. (Fig. 4).

The remaining 20 samples were scanned using Scan Xmate-L090 (Comscantecno, Yokohama, Japan) with a voxel size of 21.47 μm. CSA of the tunnel was measured at three planes (at 24, 64, and 104 mm from the entrance of the tunnel) with ImageJ 1.50i, and the ratio of the measured CSA was calculated based on an expected CSA as the CSA ratio. (rectangular tunnel was 4 × 5 = 20 mm^2^; round tunnel was 2.5 × 2.5 × 3.14 = 19.625 mm^2^).

### Statistical analysis

Statistical analysis was performed between the US and the abrader burr/drill groups using the Mann-Whitney’s U test and less than .05 indicated a significant difference.

## Results

### Curettage

**Fig. 5:**
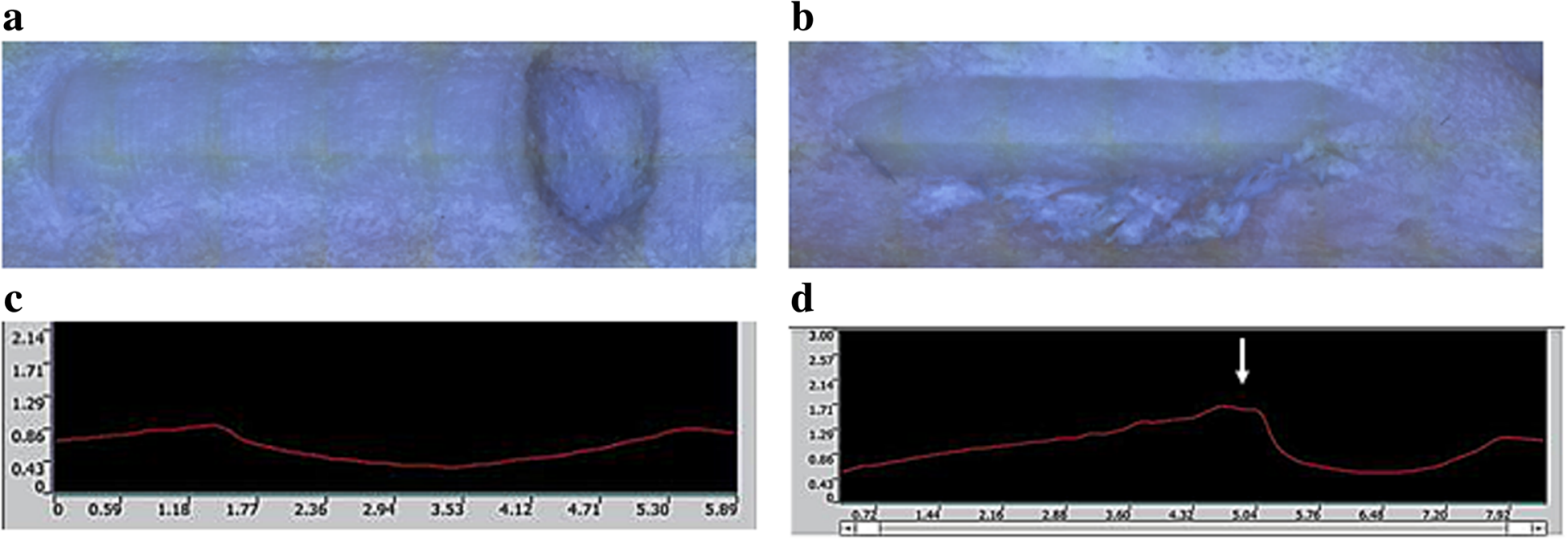
Curetted bone surface (**a**, **b**), and cross section of the gutter (**c**, **d**). **a**) and **c**) US device; **b**) and **d**) abrader burr. A bank (arrow) was observed on left side of the gutter in use of the abrader burr (**d**)

The depth of curettage and bottom angle were significantly smaller with the US device than with the abrader burr at all planes, while the curetted area ratio created by each device was mostly equal to the other. (Table 1) At each plane, debris was found at the edge of the gutter in the abrader group, while it was seldom observed in the US group. (Fig. 5) Surface roughness was significantly smaller with the US device than with the abrader burr. (Table 2).

**Table 1:**
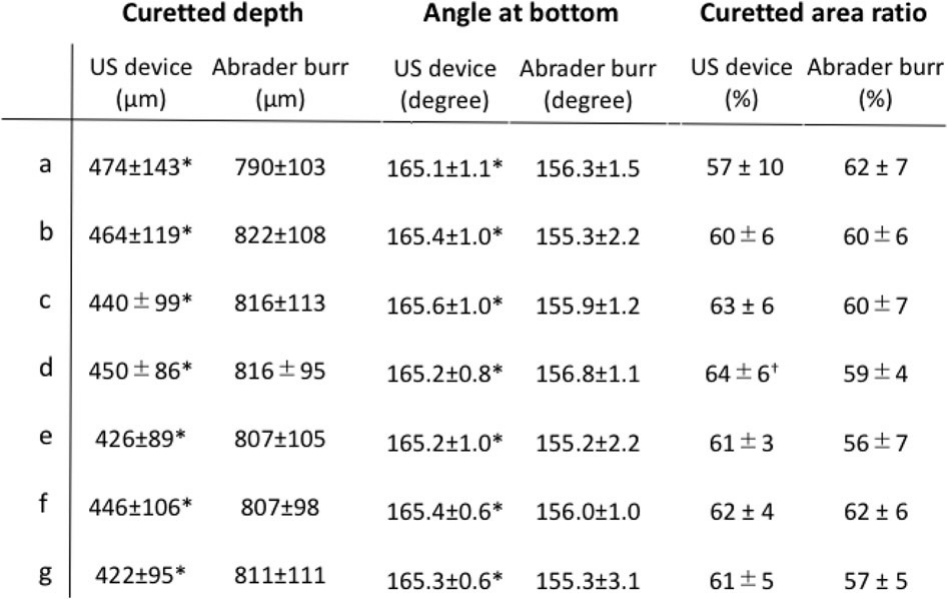
Comparison of curetted depth, angle at bottom, and curetted area ratio of the gutter between the US device and abrader burr. *: *p* < .01, +: *p* < .05

**Table 2:**
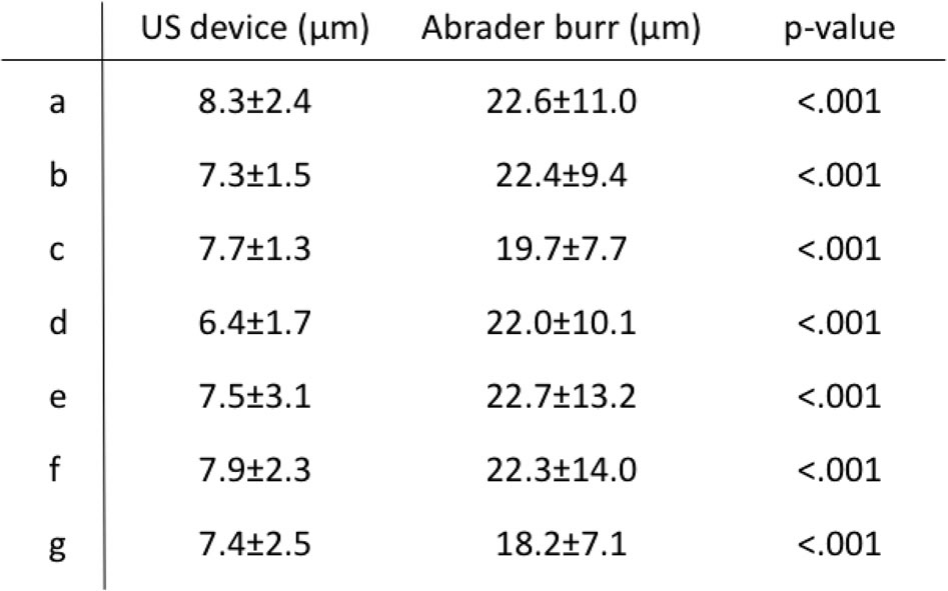
Roughness of the curetted bone surface

### Excavation

**Fig. 6:**
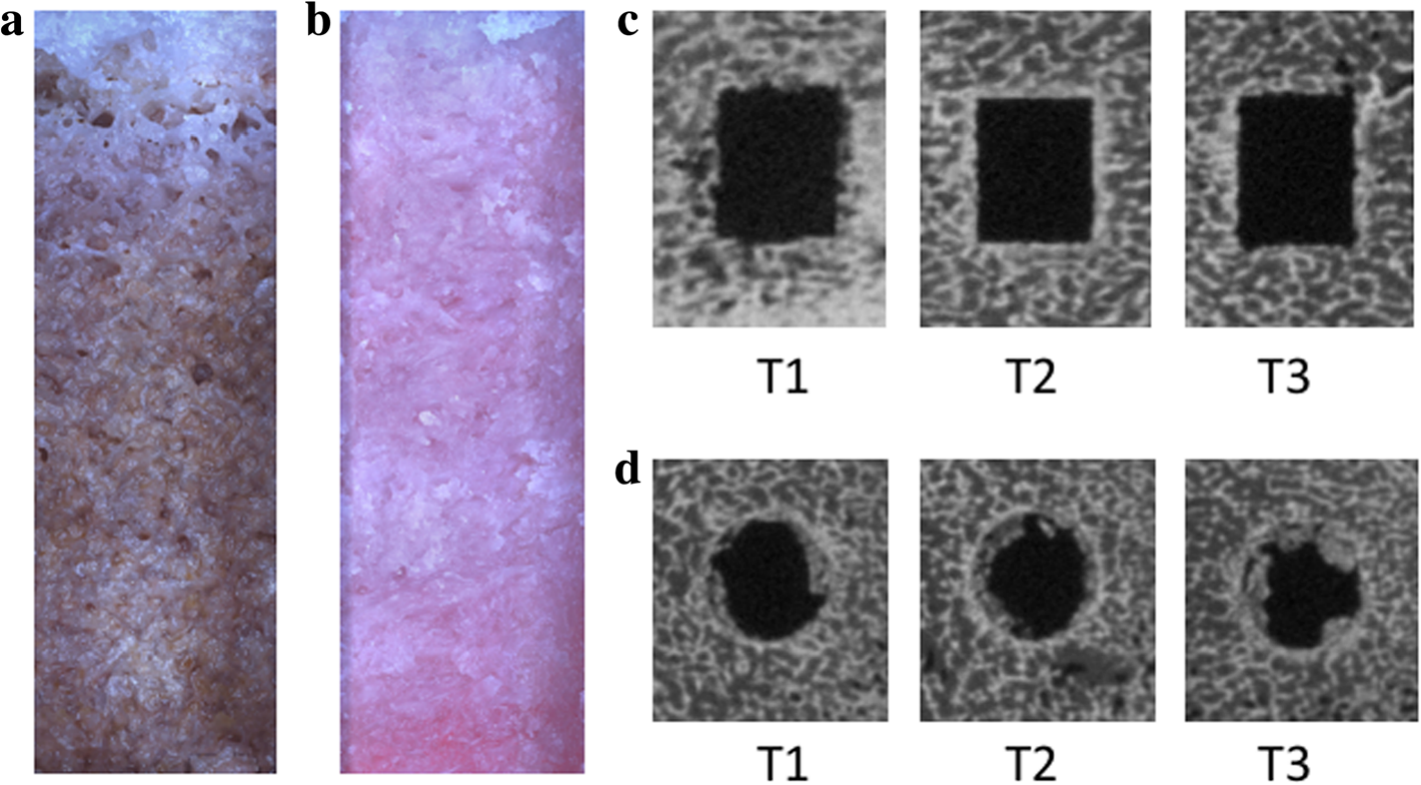
Walls and cross-sectional shape of tunnel. **a**) and **c**) rectangular blade; **b**) and **d**) Endobutton drill bit. Some bony particle was observed in the tunnel created with a drill bit

Surface and linear roughness at area A were significantly larger in US device, while there was no significant difference at the other two areas. (Table 3) CSA ratio with the US device was significantly smaller than that with the drill at each slice and was closer to 100%. (Table 4) Moreover, the cross section of the tunnel was clear with the US device, while bone particles were often observed around the walls when drilling. (Fig. 6).

**Table 3:**
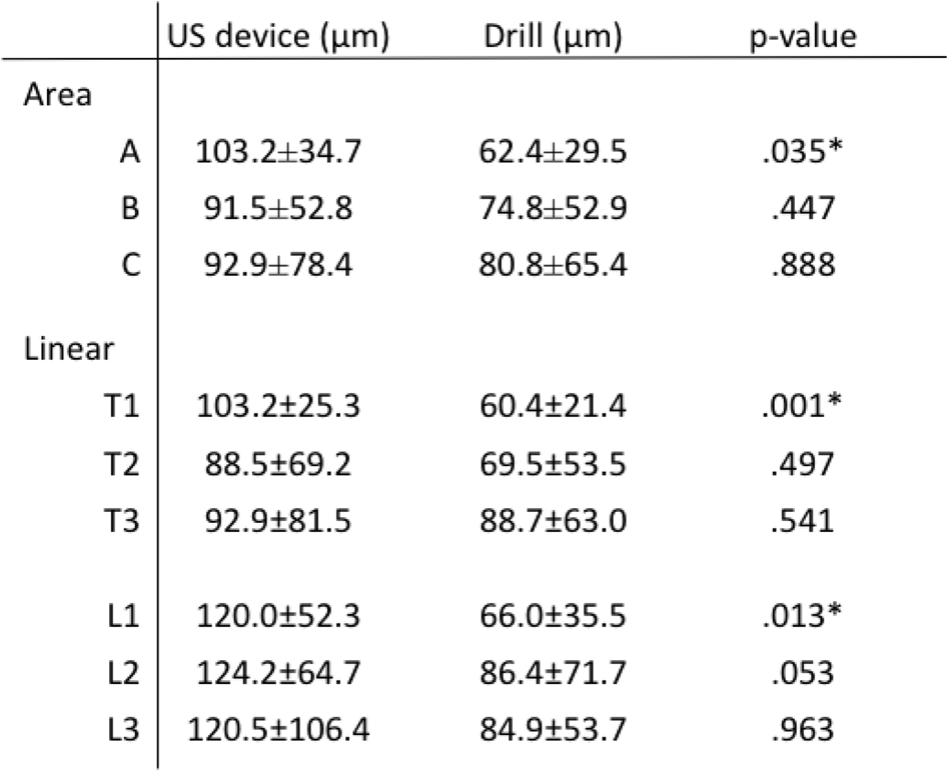
Roughness of the tunnel wall. Evaluation as area and linear line

**Table 4:**
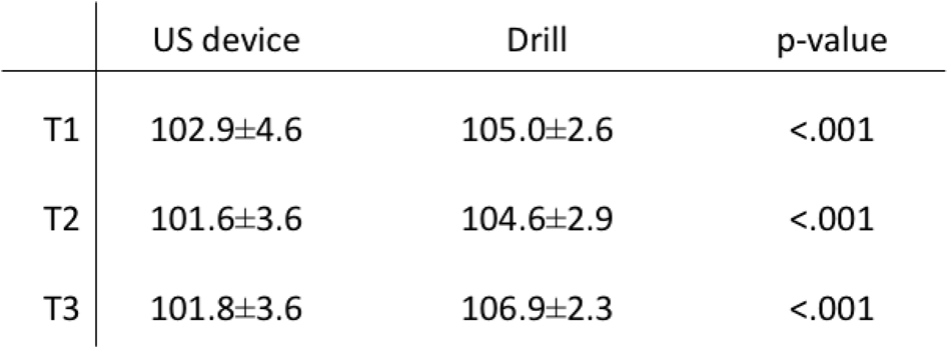
Cross-sectional area ratio of the gutter

## Discussion

The principle findings of this study were that the roughness of the curetted surface was smoother with the US device than with the abrader burr, and that the tunnel shape itself was more accurate with the US device than with the drill bit in excavation. Therefore, this US device will be advantageous in creating a smoother surface and accurate tunnel during arthroscopic surgery, compared to the conventional devices.

The US surgical device has begun to be used for osteotomy in oral surgery and the use of air-driven sonic osteotomes has been reported in some clinical studies (Agabiti, 2011; Geminiani et al., 2011; Papadimitriou et al., 2012; Vercellotti, 2000; Vercellotti, 2004). Papadimitrious et al. (Papadimitriou et al., 2012) described an alternative technique for atraumatic tooth extraction using an air-driven sonic instrument for preservation of an intact labial plate. US bone removers are also used for skull base surgery and have been introduced in the field of orthopaedic surgery as spinal surgery, such as for anterior clinoidectomy, Le Fort I osteotomy, and spinal laminectomy (Chang et al., 2006; Hadeishi et al., 2003; Hazer et al., 2016; Nakagawa et al., 2005; Timothy et al., 2018; Ueki et al., 2004). Hazer et al. (Hazer et al., 2016) reported the US bone curette to be useful in very narrow epidural spaces, while avoiding excessive heat production, minimizing blood loss and operating time, and limiting the risk of mechanical injury. Thus, they recommended the device for various spinal surgery fields and especially as the only tool for limited foraminotomies. However, US instruments for arthroscopic surgery have not been introduced and reported because of the irrigation resistance. To our knowledge, this is the first study to use an US device in water and to clarify the characteristics of the US device considered for arthroscopic surgery.

The CSA of curetted gutter with the US device was similar to that with the abrader burr. Those devices moved on cortical bone three times with 3 N of compressive load using the same custom-made machine, although the shape of the curetting device and the mechanism of curetting were different. Thus, the curetted volume of both devices was equal under the same loading condition, even though the device was different. Therefore, the curetted volume may depend on the loading condition and sharpness of the blade. On the other hand, the depth of the gutter and the angle at the bottom of the gutter were smaller with the US device. Therefore, the US device has an advantage to flatly shave the bone surface and can shave bone widely and consistently, whereas the abrader burr is suitable to effectively and sharply curette bone. Moreover, the roughness of the curetted surface was smoother with the US device than with the abrader burr. The blade of the abrader looks like a burr and rotates during bone curettage, while the tip of the US device just sweeps the bone surface. Thus, the US device is also superior in regard to smoothness. As the abrader burr removes bone by rotating in one direction, one side of the gutter has a bony hill and it takes some time to remove this hill to create a smooth surface when using the abrader burr. On the other hand, the US curettage is used by pushing or pulling in one direction, so that the bony hill is made forward or backward of the gutter. However, the height of the hill is not so great as the depth of the gutter is not deep with one curettage. Thus, we can recommend the use of this US device arthroscopically to make a smooth bony surface, though some device for outflow must be necessary to remove the debris from the US device, which has no suction function.

The US assisted drilling was previously reported and could reduce the temperature and the amount of microcracks compared to the conventional drilling (Alam & Silberschmidt, 2014; Alam et al., 2015; Scarano et al., 2014; Wang et al., 2013; Wang et al., 2014). However, no reports have clarified the CSA of the bone tunnel and the surface property. As a cannulated drill is usually moved along a guide wire, the room between a guide wire and a cannulated space in the drill bit generates play of rotation and can cause excessive bony excavation. Moreover, the tip of the drill bit may possibly rotate widely due to centrifugal force, as the rotator in the handpiece itself rotates and transmits the power to the tip of the drill bit. These are the main reasons why the measured CSA was larger than the expected CSA calculated from the diameter of the drill head. On the other hand, as the US device excavates the bone tunnel with vibration in the long axis direction, the effect of centrifugal force was small and the device created a quite accurate tunnel creation in size. However, the roughness of the tunnel surface was larger at one area with the US device, compared to the surface created with the drill. The US device does not curette bones at the side of the tunnel, but at the head of the device, while the drill moves forward with curetting bones at the side of the tunnel as well as at the front of the drill. Thus, the drill can make the smooth surface of the tunnel when using a sharp drill bit.

### Limitation

There are some limitations in this study. First, porcine bones were used, while it might have been more clinically applicable to use human bone. However, as most of the human cadaveric knees are acquired from elders and frequently exhibit osteoporosis, use of young porcine knees could reduce the influence of bone quality. Second, we evaluated only one size of the US device in each examination, as only two probes have been developed to date. The other size and type of US probes will be used in future. Third, the roughness of the surface was measured with a macroscopic scope. The roughness can be evaluated with coefficient of friction.

## Conclusions

1. US curettage has an advantage to flatly curette bone surfaces, and is superior to shaving bone widely and consistently.

2. A bone tunnel can be accurately created with the US device, while the tunnel wall is partially rough.
